# Whole Exome Analysis Identifies Frequent *CNGA1* Mutations in Japanese Population with Autosomal Recessive Retinitis Pigmentosa

**DOI:** 10.1371/journal.pone.0108721

**Published:** 2014-09-30

**Authors:** Satoshi Katagiri, Masakazu Akahori, Yuri Sergeev, Kazutoshi Yoshitake, Kazuho Ikeo, Masaaki Furuno, Takaaki Hayashi, Mineo Kondo, Shinji Ueno, Kazushige Tsunoda, Kei Shinoda, Kazuki Kuniyoshi, Yohinori Tsurusaki, Naomichi Matsumoto, Hiroshi Tsuneoka, Takeshi Iwata

**Affiliations:** 1 Division of Molecular and Cellular Biology, National Institute of Sensory Organs, National Hospital Organization Tokyo Medical Center, Tokyo, Japan; 2 Department of Ophthalmology, The Jikei University School of Medicine, Tokyo, Japan; 3 National Eye Institute, National Institutes of Health, Bethesda, MD, United States of America; 4 Laboratory of DNA Data Analysis, National Institute of Genetics, Shizuoka, Japan; 5 RIKEN Center for Life Science Technologies, Division of Genomic Technologies, Life Science Accelerator Technology Group, Transcriptome Technology Team, Yokohama, Japan; 6 Department of Ophthalmology, Mie University Graduate School of Medicine, Tsu, Japan; 7 Department of Ophthalmology, Nagoya University Graduate School of Medicine, Nagoya, Japan; 8 Laboratory of Visual Physiology, National Institute of Sensory Organs, Tokyo, Japan; 9 Department of Ophthalmology, Teikyo University School of Medicine, Tokyo, Japan; 10 Department of Ophthalmology, Kinki University Faculty of Medicine, Osaka, Japan; 11 Department of Human Genetics, Yokohama City University, Yokohama, Japan; King Faisal Specialist Hospital and Research center, Saudi Arabia

## Abstract

**Objective:**

The purpose of this study was to investigate frequent disease-causing gene mutations in autosomal recessive retinitis pigmentosa (arRP) in the Japanese population.

**Methods:**

In total, 99 Japanese patients with non-syndromic and unrelated arRP or sporadic RP (spRP) were recruited in this study and ophthalmic examinations were conducted for the diagnosis of RP. Among these patients, whole exome sequencing analysis of 30 RP patients and direct sequencing screening of all *CNGA1* exons of the other 69 RP patients were performed.

**Results:**

Whole exome sequencing of 30 arRP/spRP patients identified disease-causing gene mutations of *CNGA1* (four patients), *EYS* (three patients) and *SAG* (one patient) in eight patients and potential disease-causing gene variants of *USH2A* (two patients), *EYS* (one patient), *TULP1* (one patient) and *C2orf71* (one patient) in five patients. Screening of an additional 69 arRP/spRP patients for the *CNGA1* gene mutation revealed one patient with a homozygous mutation.

**Conclusions:**

This is the first identification of *CNGA1* mutations in arRP Japanese patients. The frequency of *CNGA1* gene mutation was 5.1% (5/99 patients). *CNGA1* mutations are one of the most frequent arRP-causing mutations in Japanese patients.

## Introduction

Retinitis pigmentosa (RP; OMIM #268000) is a heterogeneous group of inherited disorders characterized by visual field loss, night blindness, abnormal color vision and fundus degeneration. The prevalence of RP is approximately 1 per 4,000 persons and more than 1 million individuals are affected worldwide [1]. The inheritance of RP shows various patterns including autosomal recessive (arRP), autosomal dominant, X-linked, sporadic (spRP), mitochondrial [2] and digenic [3] inheritance. Among the various patterns of RP inheritance, arRP is the most frequent inheritance pattern and accounts for approximately 50% to 60% of all RP patients [1]. To date, 42 arRP-causing genes and three loci have been reported in the Retinal Information Network (RetNet; https://sph.uth.edu/retnet/). Among these arRP-causing genes, mutations in Usher syndrome 2A (*USH2A*) are the most frequent and account for approximately 17% cases including cases with additional hearing loss [1]. In non-syndromic arRP, the most frequent arRP genes are eyes shut homolog (*EYS*), *USH2A* and ATP-binding cassette sub-family A member 4 (*ABCA4*), which account for approximately 10%, 8% and 5% to 6% of cases, respectively [1], [4]–[10].

Large scale screening of selective exons in 30 RP-causing genes was previously performed in 193 Japanese RP families [11]. Although it only targeted exons with known mutations, the study failed to identify high frequency RP genes [11]. Another study of the Japanese RP population focused on the RP genes *RHO* [12]–[14] and *EYS* [15], [16], and found that *EYS* was a frequent arRP gene with a prevalence rate of 9% to 16% [15], [16]. However, almost all reported *EYS* gene mutations in these studies have not been reported in Western populations suggesting that Japanese individuals have a different genetic background [15], [16]. These results suggest that the genetic background of RP in the Japanese population is different from that in the Western population.

The recent technological development of exon capture with 99% coverage of all exons and its combination with next generation sequencing enables effective genetic studies for hereditary diseases [17]–[20] and the investigation of novel mutations in multiple candidate genes [21].

The purpose of this study was to find frequent arRP genes in the Japanese population. In this study, we performed whole exome analysis of 30 Japanese arRP/spRP patients with confirmation in an additional 69 arRP/spRP patients. We found frequent arRP-causing mutations in the cyclic nucleotide gated channel alpha 1 (*CNGA1*) gene.

## Materials and Methods

### Informed consent

The protocol of this study was approved by the Institutional Review Board at the six participating institutions (National Hospital Organization Tokyo Medical Center, Jikei University School of Medicine, Mie University School of Medicine, Nagoya University Graduate School of Medicine, Teikyo University School of Medicine and Kinki University Faculty of Medicine). The protocol adhered to the tenets of the Declaration of Helsinki, and signed informed consent was obtained from all participants.

### Clinical studies

In total, 99 unrelated arRP/spRP patients with no apparent syndrome were recruited from the National Hospital Organization Tokyo Medical Center, Jikei University School of Medicine, Mie University School of Medicine, Nagoya University Graduate School of Medicine, Teikyo University School of Medicine and Kinki University Faculty of Medicine. The patient history was taken and ophthalmic examinations were performed. Clinical diagnosis and evaluation for RP were based on the decimal best-corrected visual acuity (BCVA), slit-lamp examination, fundus examination, visual fields determined using kinetic perimetry (Goldmann perimeter [GP]; Haag Streit, Bern, Switzerland) and electroretinography (ERG) findings. Characteristic findings for diagnosis of RP include progressive visual field loss from peripheral, night blindness, abnormal color vision, fundus degeneration represented by bone spicule pigmentations and attenuation of retinal vessels, and the more or equally decreased rod responses compared with cone responses of ERG [1], [22].

### DNA preparation and exome sequencing analysis

We obtained venous blood samples from all participants and genomic DNA was extracted. Whole exome sequencing was performed for 30 arRP/spRP patients using a method previously described [23]. Briefly, construction of paired-end sequence libraries and exome capture were performed by using the Agilent Bravo automated liquid-handling platform with SureSelect XT Human All Exon kit V4+ UTRs kit (Agilent Technologies, Santa Clara, CA). Enriched libraries were sequenced by using an Illumina HiSeq2000 sequencer. Reads were mapped to the reference human genome (1000 genomes phase 2 reference, hs37d5) with Burrows–Wheeler Aligner software version 0.6.2 [24]. Duplicated reads were then removed by Picard Mark Duplicates module version 1.62, and mapped reads around insertion/deletion polymorphisms were realigned by using the Genome Analysis Toolkit (GATK) version 2.1–13 [25]. Base-quality scores were recalibrated by using GATK. To extract potentially RP-causing variants, we focused only on variants that could change the amino acid sequence, such as non-synonymous variants, splice acceptor and donor site variants, and insertion/deletion polymorphisms. The identified variants were filtered by a frequency of less than 1% in the 1000 Genomes project (http://www.1000genomes.org) and the Human Genetic Variation Browser (http://www.genome.med.kyoto-u.ac.jp/SnpDB/about.html). The remained variants were further screened within 212 genes registered as retinal disease-causing genes in the RetNet database updated on March 10, 2014. All remained variants of 30 arRP/spRP patients were summarized in Table S1 in File S1. Selection of disease-causing mutations was restricted to three genetic criteria: first, homozygosity or compound heterozygosity of known arRP-causing mutations; second, compound heterozygosity of known and predicted arRP-causing mutations; and third, homozygosity or compound heterozygosity of predicted arRP-causing mutations. Mutations were defined as disease causing only if these criteria were fulfilled. Mutations causing exon truncation through frameshift, splicing and termination were considered to be more severe than missense mutations with unknown pathogenic relevance. In addition, to investigate the potential disease-causing variants, we added three genetic criteria: first, compound heterozygosity of known arRP-causing mutation and missense potential arRP-causing variant; second, compound heterozygosity of predicted arRP-causing mutation and potential arRP-causing variant; and third, homozygosity or compound heterozygosity of potential arRP-causing variants.

### Direct sequencing of the *CNGA1* gene

The *CNGA1* mutations identified by whole exome sequencing were further confirmed by direct sequencing. An additional 69 arRP/spRP patients were analyzed by direct sequencing for all coding exons (4 to 11) of *CNGA1*. The targeted exons (4 to 11) of the *CNGA1* gene were amplified by PCR using the primer pairs given in Table S2 in File S1. The PCR products were purified using Agencourt APMure XP (Beckman Coulter, Brea, CA) and used as a template for sequencing. Both DNA strands were sequenced by an automated sequencer (3730*xl* DNA Analyzer; Life Technologies Corporation, Carlsbad, CA) using the BigDye Terminator kit V3.1 (Life Technologies Corporation).

### Assessment of found mutations or variants in this study

Novel mutations and variants were defined as those not present in the literature, dbSNP database (http://www.ncbi.nlm.nih.gov/SNP/), Human Genetic Variation Browser, 1000 Genome project database or the Human Gene Mutation Database (http://www.hgmd.cf.ac.uk). In addition, the frequency of identified mutations or variants in this study was investigated using in-house exome sequencing data from 575 unaffected Japanese controls at Yokohama City University. Segregation was confirmed for both the arRP-causing mutations and potential arRP-causing variants by direct sequencing when parent samples were available.

## Results

### Whole exome sequencing analysis and identification of frequent arRP gene mutations

To identify frequent arRP-causing genes, we performed whole exome sequencing in non-syndromic 30 arRP/spRP patients. We focused on 212 retinal disease-causing genes registered in RetNet database updated on March 10, 2014. The average of mean depth for all 30 samples reached 71.11±7.68-fold and the average of coverage at 4- and 12-fold for all 30 samples reached 98.1% and 92.5% respectively. The analysis of arRP-causing mutations and potential arRP-causing variants was conducted according to the criteria described in Materials and Methods. Segregation of identified arRP-causing mutations and potential arRP-causing variants were conducted in five families: RP#002, RP#004, RP#011, RP#016 and RP#019. Although the results of segregation in RP#002, RP#004, RP#016 and RP#019 matched the inheritance pattern, two *USH2A* variants in RP#011 (Table S1 in File S1) did not match the inheritance pattern because the father of RP#011 carried two identical *USH2A* variants. Therefore, we concluded that the two *USH2A* variants in RP#011 were not arRP-causing. Consequently, the exome analysis identified eight arRP-causing mutations including three novel mutations and five known mutations in eight arRP/spRP patients [6], [16], [26], [27] and identified potential arRP-causing variants including six novel variants and five known variants in five arRP/spRP patients (Table 1). The arRP-causing mutations were found in *CNGA1* (four patients), *EYS* (three patients) and S-antigen retina and pineal gland (*SAG*) (one patient). Potential arRP-casing variants were found in *USH2A* (two patients), *EYS* (one patient), tubby like protein 1 (*TULP1*) (one patient) and chromosome 2 open reading frame 71 (*C2orf71*) (one patient). Among these genes, the most frequent arRP-causing gene was *CNGA1*. In particular, pedigree RP#002 with a homozygous c.191delG (p.G64VfsX29) mutation, RP#019 with compound heterozygous c.265delC (p.L89FfsX4) and c.1429delG (p.V477YfsX17) mutations, RP#021 with a homozygous c.191delG mutation and RP#029 with a homozygous c.265delC mutation were identified. Further direct sequencing confirmed that the parents in pedigree RP#019 had c.265delC or c.1429delG respectively. The *CNGA1* sequence was compared with the NCBI reference sequence for the *CNGA1* transcript (GenBank ID; NM_000087.3).

**Table 1:** Autosomal recessive retinitis pigmentosa (arRP)-causing mutations and potential arRP-causing variants found by exome sequencing.

Family ID	Gene Name	GenBank ID	Exon	Nucleotide Change	Amino Acid Change	State	Frequency*	SNP ID	Reference	Pathogenicity
RP#002	*CNGA1*	NM_000087	5	c.191delG	p.G64VfsX29	Homo	2		HGVB	Disease-causing
RP#004	*EYS*	NM_001142800	33	c.6714delT	p.P2238PfsX16	Hetero	0		Collin et al. 2008	Disease-causing
	*EYS*	NM_001142800	35	c.C7002A	p.C2334X	Hetero	0		This study	
RP#014	*EYS*	NM_001142800	4	c.A141T	p.E47D	Hetero	0		This study	Potential disease-causing
	*EYS*	NM_001142800	26	c.4957dupA	p.S1653KfsX2	Hetero	2		Iwanami et al. 2012	
RP#016	*TULP1*	NM_003322	1	c.G3A	p.M1I	Hetero	0		This study	Potential disease-causing
	*TULP1*	NM_003322	13	c.C1246T	p.R416C	Hetero	0	rs200769197	dbSNP	
RP#017	*EYS*	NM_001142800	26	c.4022delC	p.S1341FfsX11	Hetero	0		This study	Disease-causing
	*EYS*	NM_001142800	26	c.4957dupA	p.S1653KfsX2	Hetero	2		Iwanami et al. 2012	
RP#019	*CNGA1*	NM_000087	6	c.265delC	p.L89FfsX4	Hetero	2		Chen et al. 2013	Disease-causing
	*CNGA1*	NM_000087	11	c.1429delG	p.V477YfsX17	Hetero	0		This study	
RP#021	*CNGA1*	NM_000087	5	c.191delG	p.G64VfsX29	Homo	2		HGVB	Disease-causing
RP#023	*USH2A*	NM_206933	49	c.C9676T	p.R3226X	Hetero	0		This study	Potential disease-causing
	*USH2A*	NM_206933	55	c.T10859C	p.I3620T	Hetero	0		HGVB	
RP#026	*EYS*	NM_001142800	26	c.4957dupA	p.S1653KfsX2	Homo	2		Iwanami et al. 2012	Disease-causing
RP#027	*SAG*	NM_000541	11	c.926delA	p.T309TfsX12	Homo	6		Fuchs et al. 1995	Disease-causing
RP#028	*USH2A*	NM_206933	41	c.T7880C	p.I2627T	Hetero	0		This study	Potential disease-causing
	*USH2A*	NM_206933	55	c.C10931T	p.T3644M	Homo	1	rs185823130	dbSNP	
	*USH2A*	NM_206933	70	c.T15178C	p.S5060P	Hetero	0		This study	
RP#029	*CNGA1*	NM_000087	6	c.265delC	p.L89FfsX4	Homo	2		Chen et al. 2013	Disease-causing
RP#030	*C2orf71*	NM_001029883	1	c.C85T	p.R29W	Hetero	4	rs201706430	dbSNP	Potential disease-causing
	*C2orf71*	NM_001029883	2	c.C3748T	p.R1250C	Hetero	0		This study	

HGVB = Human Genetic Variation Browser (http://www.genome.med.kyoto-u.ac.jp/SnpDB/about.html); dbSNP = (http://www.ncbi.nlm.nih.gov/SNP/); Frequency* show the number of mutations or variants found in 1150 alleles of 575 controls.

### Screening of all *CNGA1* exons in 69 additional arRP/spRP Japanese patients

**Figure 1:**
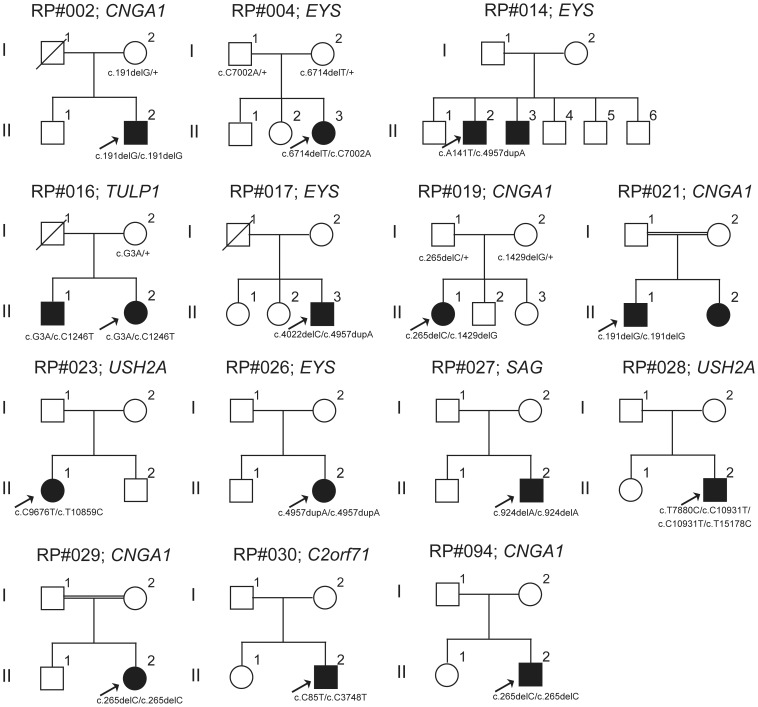
Pedigrees identified with arRP-causing mutations or potential arRP-causing variants. The solid squares (male) and circles (female) represent affected patients. The proband of each family is indicated by a black arrow. Unaffected family members are represented by white icons. The slash symbol indicates deceased individuals. The doubled line indicates consanguineous marriage. The generation number is shown on the left.

Direct sequencing of the coding region of the *CNGA1* gene in 69 arRP/spRP patients identified homozygous c.265delC mutation in pedigree RP#094 and three heterozygous variants c.G860A (p.R287K), c.G1271A (p.R424Q) and c.G2042C (p.G681A) in pedigrees RP#040, RP#063 and RP#087 respectively. All pedigrees identified to have arRP-causing mutations or potential arRP-causing variants are shown in Figure 1.

### Identified *CNGA1* mutations and variants

**Figure 2:**
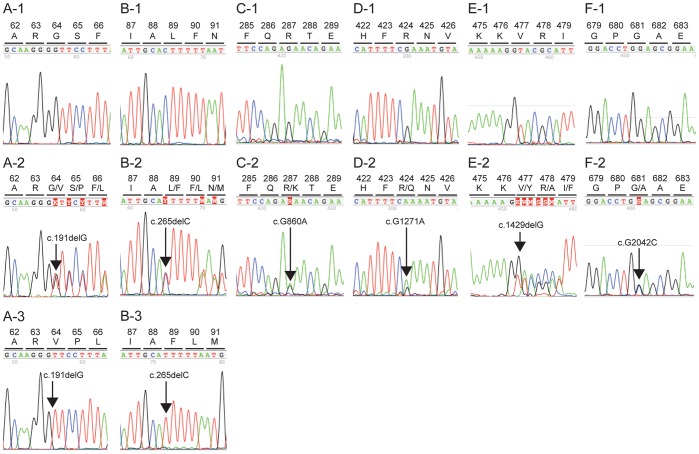
Sequence data of all six identified *CNGA1* mutations in this study. A-1 to F-1 show the normal sequence data for the *CNGA1* gene. A-2 to F-2 show the sequence data for heterozygous *CNGA1* mutations (c.191delG, c.265delC, c.G860A, c.G1271A, c.1429delG and c.G2042C, respectively). A-3 and B-3 show the sequence data for homozygous *CNGA1* mutations (c.191delG and c.265delC).

Among the three arRP-causing mutations and three variants found in this study, two (c.265delC and c.G1271A) were previously reported as arRP-causing or potential arRP-causing [11], [26] and four were not reported as arRP-causing or potential arRP-causing (c.191delG, c.265delC, c.G860A and c.G2042C). The polyphen-2 program predicted that all three missense variants in p.R287K (c.G860A), p.R424Q (c.G1271A) and p.G681A (c.G2042C) were benign. In contrast, the SIFT program predicted that p.R424Q (c.G1271A) potentially could cause severe damage to the protein, whereas p.G681A (c.G2042C) and p.R287K (c.G860A) potentially could cause mild damage. All identified mutations and variants in *CNGA1* gene are summarized in Table 2 and sequence data are given in Figure 2.

**Table 2:** Identification of patients with *CNGA1* sequence mutations and variants in this study.

Family ID	Exon	Nucleotide Change	Amino Acid Change	State	Frequency*	Polyphen-2 (score)	SIFT (score)	SNP ID	Reference	Pathogenicity
RP#002	5	c.191delG	p.G64Vfs29X	Homo	2				HGVB	Disease-causing
RP#019	6	c.265delC	p.L89FfsX4	Hetero	2				Chen et al. 2013	Disease-causing
	11	c.1429delG	p.V477YfsX17	Hetero	0				This study	
RP#021	5	c.191delG	p.G64Vfs29X	Homo	2				HGVB	Disease-causing
RP#029	6	c.265delC	p.L89FfsX4	Homo	2				Chen et al. 2013	Disease-causing
RP#040	11	c.G1271A	p.R424Q	Hetero	7	Benign (0.266)	Damaging (0)	rs192912733	Jin et al. 2008	Not disease-causing
RP#063	11	c.G2042C	p.G681A	Hetero	1	Benign (0.001)	Tolerated (0.36)		HGVB	Not disease-causing
RP#087	11	c.G860A	p.R287K	Hetero	5	Benign (0.101)	Tolerated (0.32)		HGVB	Not disease-causing
RP#094	6	c.265delC	p.L89FfsX4	Homo	2				Chen et al. 2013	Disease-causing

Polyphen-2 (http://genetics.bwh.harvard.edu/pph2/); SIFT (http://sift.jcvi.org); HGVB = Human Genetic Variation Browser (http://www.genome.med.kyoto-u.ac.jp/SnpDB/about.html); Frequency* show the number of mutations or variants found in 1150 alleles of 575 controls.

### Haplotype analysis

The haplotypes of *CNGA1* and the surrounding sequences were determined for four arRP patients, RP#002, RP#019, RP#021 and RP#029. Single-nucleotide polymorphisms (SNPs) with a frequency higher than 5% (1000 Genomes project database) were determined within 1 kb upstream and downstream of *CNGA1* (chromosome 4, positions 47,937,994–48,014,961) as shown in Table S3 in File S1. The haplotype analysis determined an identical haplotype for four alleles in patients RP#002 and RP#021 suggesting a common ancestor for the c.191delG mutation. Moreover, identical haplotypes for one allele in patient RP#019 and for both alleles in patient RP#029 were detected suggesting a common ancestor for the c.265delC mutation.

### Clinical features of *CNGA1* mutations

**Figure 3:**
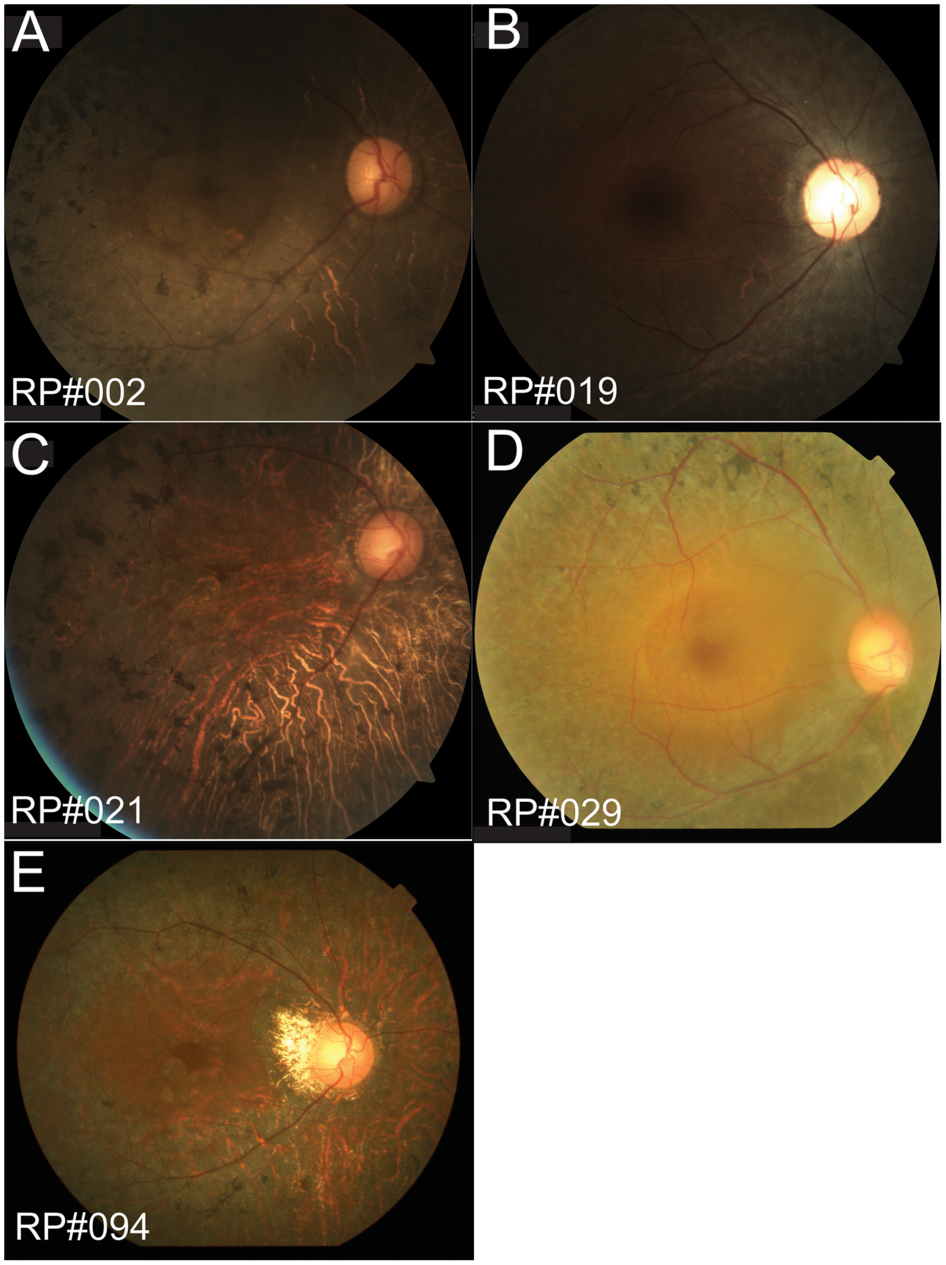
Fundus photographs of the patients with heterozygous or homozygous *CNGA1* mutations. Funduscopy indicates retinal degeneration with pigmentation and attenuation of retinal vessels in all patients. Macular edema does not existed in any patient, although retinal degeneration in the macular region is observed in RP#002, RP#021 and RP#094 (A, C and E).

To characterize the clinical features of patients with *CNGA1* mutations, we additionally investigated the clinical data of five patients with compound heterozygous or homozygous *CNGA1* mutations (Table 3). All five patients reported that they noticed night blindness from childhood. Funduscopy showed retinal degeneration with pigmentation and attenuation of retinal vessels in all patients (Fig. 3). Macular edema was not observed in any patients, although retinal degeneration in macular regions was detected in RP#002, RP#021 and RP#094 (Fig. 3A, 3C and 3E). The BCVA of RP#019 and RP#029 remained at 1.0, whereas that of RP#002, RP#021 and RP#094 was reduced. ERG showed no recordable pattern in four patients and could not be conducted in one patient. The GP of RP#022 showed ring scotoma with a preserved peripheral visual field, whereas that of another four patients was severely constricted.

**Table 3:** Ophthalmic findings in five patients with retinitis pigmentosa with compound heterozygous or homozygous *CNGA1* mutations.

Patient	Diagnosed Age, Examined Age, Sex	Onset of night blindness	BCVA		ERG	Visual field	Mutations
			Right	Left			
RP#002	42, 51, M	Childhood	0.5	0.7	Non-recordable	Severely constricted	c.191delG/c.191delG
RP#019	26, 35, F	Childhood	1.0	1.0	Non-recordable	Ring scotoma	c.265delC/c.1429delG
RP#021	60, 65, M	Childhood	0.2	0.1	Non-recordable	Severely constricted	c.191delG/c.191delG
RP#029	25, 51, F	Childhood	1.0	1.0	Non-recordable	Severely constricted	c.265delC/c.265delC
RP#094	16, 46, M	Childhood	0.4	0.3	Non-recordable	Severely constricted	c.265delC/c.265delC

BCVA = decimal corrective visual acuity; ERG = electroretinography; M = male; F = female.

## Discussion

Mutations in the *CNGA1* gene were identified for the first time in a Japanese population with a high frequency of 5.1% for homozygous or compound heterozygous mutations. The exome analysis of arRP/spRP patients revealed that 43.3% carried arRP-causing mutations or potential arRP-causing variants in *CNGA1* (13.3%), *EYS* (13.3%), *USH2A* (6.7%), *C2orf71* (3.3%), *SAG* (3.3%) and *TULP1* (3.3%). Although the prevalence of other five gene mutations was consistent with that in previous studies [1], [4]–[8], [28]–[30], the prevalence of *CNGA1* was clearly higher than in population of European descent [31], [32]. We screened for mutation in all the coding exons of *CNGA1* in an additional 69 arRP/spRP Japanese patients to further investigate the prevalence of *CNGA1* mutations in the Japanese population. We identified an arRP-causing homozygous *CNGA1* mutation in one patient. Consequently, three *CNGA1* frameshift mutations (c.191delG, c.265delC and c.1429delG) were identified as arRP-causing mutations in five patients (Table 2).

Rod cyclic nucleotide-gated ion channels contain CNGA1 and CNGB1 protein at a ratio of 3 CNGA1∶1 CNGB1 [33]. Each molecule of CNGA1 protein has at least three functional domains as described in the UniProtKB (acc. # P29973, http://www.uniprot.org, Cross-References, ProteinModelPortal); these domains function as a cation-transporter domain (residues 202–396, the Pfam ion_trans motif, http://pfam.sanger.ac.uk), cGMP-binding domain (residues 404–596, SWISSMODEL structure based on the PDB file: 4hbn_A, http://www.rcsb.org/pdb/home/home.do) and carboxy-terminal leucine zipper (CLZ) domain (residues 623–690, experimental structure based on the PDB file:3swf). The p.G64VfsX29 (c.191delG) and p.L89FfsX4 (c.265delC) protein had no transmembrane lesions, and most of the protein structure including all three functional domains was abolished. In contrast, the p.V477YfsX17 (c.1429delG) mutant protein had the correct structure up to 5th transmembrane domain helix, but lacked the 6th transmembrane domain helix, the cGMP-binding site, and the coiled-coil CLZ domain. The cGMP-binding site is important for the function of CNGA1 as a cation channel. Loss of the cGMP-binding site is likely to influence the final stage of the photo transduction pathway [31]. In addition, the absence of the coiled-coil CLZ domain completely disrupts the 3∶1 stoichiometry in CNG channels [33]. Although the p.V477YfsX17 (c.1429delG) mutant may retain part of its structure, the protein function is predicted to be completely lost.

We additionally identified the heterozygous *CNGA1* missense variants c.G860A (p.R287K), c.G1271A (p.R424Q) and c.G2042C (p.G681A) (Table 2). Heterozygous c.G1271A variant has been previously reported [11]. Based on the mild score given by the polyphen-2 program and the severe score given by the SIFT program, we also predicted that this variant is potentially disease causing. In contrast, the two novel missense variants c.G860A and c.G2042C were predicted to cause mild damage by both the polyphen-2 and SIFT programs suggesting that it is non-pathogenic. Overall, all three missense *CNGA1* variants (c.G860A, c.G1271A and c.G2042C) were found in only one allele of *CNGA1*. We conclude that these three *CNGA1* variants were not disease causing in nature, at least from the phenotypic observation.

The clinical course of the five patients with compound heterozygous or homozygous *CNGA1* mutations included night blindness from childhood, visual field loss in middle age, non-recordable ERG and characteristic retinal degeneration pattern of RP, which were consistent with previously reported phenotypes of *CNGA1* mutations [32], [34]. Retinal degeneration in the macular region and severely decreased BCVA occurred in 3/5 patients suggesting that the advanced stage of *CNGA1* mutations included degeneration of the entire retina with both rod and cone photoreceptors. Although the genotype-phenotype correlation for *CNGA1* mutations was not clear in this study, all five patients with *CNGA1* mutations showed typical phenotypes of RP.

Previous reports have shown a strong association of *CNGA1* with arRP [11], [26], [31], [32], [34], [35]. Dryja et al. estimated the prevalence of *CNGA1* mutations in arRP patients to be between 1.7 and 2.3% (3 or 4 of 173 patients) [31]. The prevalence of *CNGA1* mutations in a Spanish arRP population was 2.1% (1 of 46 patients) [32], whereas that in a Chinese population with hereditary retinal dystrophy was 4.0% (1 of 25 patients) [26]. The average prevalence of *CNGA1* mutations in arRP/spRP patients was 7.6% (1 of 13 patients) [26]. These findings suggest that the prevalence of *CNGA1* mutations is higher in Asian population than in populations of European descent. The prevalence of *CNGA1* mutations in Chinese populations requires further study because only one Chinese patient has been reported to have a homozygous mutation in this gene [26]. Jin et al. investigated *CNGA1* exons 6, 8 and partial 11 in 193 Japanese RP families and found a single heterozygous *CNGA1* variant (c.1271G>A) [11]. In our study, all coding exons of *CNGA1* were screened and the estimate prevalence of *CNGA1* mutations reached at 5.1% (5 of 99 patients) including four homozygous and one compound heterozygous patients. Our findings suggest that the prevalence of *CNGA1* mutations is higher in Asian populations than in European populations. Moreover, c.191delG mutation has only been reported in Human Genetic Variation Browser (the database of genetic variations in Japanese population, http://www.genome.med.kyoto-u.ac.jp/SnpDB/), c.265delC mutation only reported in Chinese population [26] and c.1429delG mutation identified as novel. The *CNGA1* mutations found in this study only overlapped with mutations identified in studies of Asian individuals indicating that the founder is specific to Asian populations. Lastly, the haplotypes for the *CNGA1* mutations found in this study were individually unique (Table S3 in File S1). Further investigation of haplotypes is required to clarify the origin of these *CNGA1* mutations.

## Supporting Information

File S1 **Supporting Tables.** Table S1, All rare variants of 30 arRP/spRP paients of this study, focusing on 212 retinal disease-causing genes registered in the Retinal Information Network (https://sph.uth.edu/retnet/). Table S2, *CNGA1* primers and PCR conditions. Table S3, Haplotype analysis of four retinitis pigmentosa patients with *CNGA1* mutations.
(DOC)
