# Supplementary material for: Whole Exome Analysis Identifies Frequent CNGA1 Mutations in Japanese Population with Autosomal Recessive Retinitis Pigmentosa
Source: PLoS One. 2014 Sep 30;9(9):e108721. doi: 10.1371/journal.pone.0108721 (PMC4182560; doi:10.1371/journal.pone.0108721)
Supplement: File S1 — Supporting Tables. Table S1, All rare variants of 30 arRP/spRP paients of this study, focusing on 212 retinal disease-causing genes registered in the Retinal Information Network (https://sph.uth.edu/retnet/). Table S2, CNGA1 primers and PCR conditions. Table S3, Haplotype analysis of four retinitis pigmentosa patients with CNGA1 mutations. (DOC) [file pone.0108721.s001.doc]

**Supplemental Table S1.**

**All rare variants of 30 arRP/spRP patients of this study, focusing on 212 retinal disease-causing genes registered in the Retinal Information Network (**[**https://sph.uth.edu/retnet/**](https://sph.uth.edu/retnet/)**)**

| RP#001 |  |  |  |  |  |  |  |
| --- | --- | --- | --- | --- | --- | --- | --- |
| Chrom | Position | Ref | Alt | Gene Name | Gene Bank ID:Exon:Nucleotide change:Amino Acid change | State | SNP ID |
| 2 | 182403891 | G | C | *CERKL* | NM_001030311:exon13:c.C1544G:p.T515S | Ref/Alt |  |
| 3 | 129252449 | A | G | *RHO* | NM_000539:exon5:c.937-2A>G | Ref/Alt |  |
| 4 | 6290790 | T | G | *WFS1* | NM_006005:exon4:c.T392G:p.V131G | Ref/Alt |  |
| 4 | 6302756 | G | C | *WFS1* | NM_006005:exon8:c.G1234C:p.V412L | Ref/Alt | rs149865710 |
| 4 | 15559009 | A | G | *CC2D2A* | NM_001080522:exon22:c.A2708G:p.Q903R | Ref/Alt |  |
| 4 | 16010678 | G | A | *PROM1* | NM_006017:exon11:c.C1195T:p.R399C | Ref/Alt |  |
| 4 | 187117196 | A | G | *CYP4V2* | NM_207352:exon3:c.A367G:p.M123V | Ref/Alt | rs149684063 |
| 5 | 82836597 | G | A | *VCAN* | NM_004385:exon8:c.G7775A:p.G2592E | Ref/Alt |  |
| 14 | 88904442 | C | A | *SPATA7* | NM_018418:exon12:c.C1476A:p.F492L | Ref/Alt |  |
| 16 | 16263649 | G | A | *ABCC6* | NM_001171:exon22:c.C2849T:p.A950V | Ref/Alt | rs190557767 |
|  |  |  |  |  |  |  |  |
| RP#002 |  |  |  |  |  |  |  |
| Chrom | Position | Ref | Alt | Gene Name | Gene Bank ID:Exon:Nucleotide change:Amino Acid change | State | SNP ID |
| 2 | 56108764 | C | T | *EFEMP1* | NM_001039348:exon6:c.G623A:p.R208Q | Ref/Alt |  |
| 2 | 73747095 | G | A | *ALMS1* | NM_015120:exon11:c.G9730A:p.A3244T | Ref/Alt | rs200432874 |
| 4 | 47953414 | AC | A | *CNGA1* | NM_000087:exon5:c.191delG:p.G64fs | Alt/Alt |  |
| 5 | 90086965 | A | G | *GPR98* | NM_032119:exon70:c.A14319G:p.I4773M | Ref/Alt | rs201953675 |
| 6 | 65303078 | A | C | *EYS* | NM_001142800:exon25:c.T3809G:p.V1270G | Ref/Alt |  |
| 17 | 36492878 | G | A | *GPR179* | NM_001004334:exon4:c.C1210T:p.R404C | Ref/Alt | rs147753316 |
|  |  |  |  |  |  |  |  |
| RP#003 |  |  |  |  |  |  |  |
| Chrom | Position | Ref | Alt | Gene Name | Gene Bank ID:Exon:Nucleotide change:Amino Acid change | State | SNP ID |
| 4 | 22389702 | C | T | *GPR125* | NM_145290:exon19:c.G3592A:p.V1198M | Ref/Alt |  |
| 5 | 82816229 | G | A | *VCAN* | NM_004385:exon7:c.G2104A:p.E702K | Ref/Alt | rs201590497 |
| 6 | 42689619 | T | C | *PRPH2* | NM_000322:exon1:c.A454G:p.M152V | Ref/Alt | rs146703538 |
| 8 | 55533999 | T | G | *RP1* | NM_006269:exon2:c.T473G:p.V158G | Ref/Alt |  |
| 10 | 73498261 | A | G | *CDH23* | NM_022124:exon33:c.A4216G:p.I1406V | Ref/Alt | rs192459984 |
| 11 | 17531103 | T | G | *USH1C* | NM_153676:exon18:c.A1813C:p.I605L | Ref/Alt |  |
|  |  |  |  |  |  |  |  |
| RP#004 |  |  |  |  |  |  |  |
| Chrom | Position | Ref | Alt | Gene Name | Gene Bank ID:Exon:Nucleotide change:Amino Acid change | State | SNP ID |
| 1 | 10042628 | C | T | *NMNAT1* | NM_022787:exon5:c.C709T:p.R237C | Ref/Alt |  |
| 1 | 19547297 | A | C | *EMC1* | NM_015047:exon21:c.T2633G:p.L878R | Ref/Alt | rs202180622 |
| 1 | 211654669 | A | T | *RD3* | NM_183059:exon2:c.T89A:p.M30K | Ref/Alt |  |
| 4 | 6296897 | A | C | *WFS1* | NM_006005:exon7:c.A842C:p.D281A | Ref/Alt | rs200841930 |
| 5 | 178410178 | G | T | *GRM6* | NM_000843:exon9:c.C2169A:p.S723R | Ref/Alt |  |
| 6 | 64694329 | G | T | *EYS* | NM_001142800:exon35:c.C7002A:p.C2334X | Ref/Alt |  |
| 6 | 64776241 | TA | T | *EYS* | NM_001142800:exon33:c.6714delT:p.P2238fs | Ref/Alt |  |
| 8 | 10466998 | G | T | *RP1L1* | NM_178857:exon4:c.C4610A:p.A1537E | Ref/Alt |  |
| 10 | 73326515 | C | T | *CDH23* | NM_022124:exon7:c.C446T:p.T149M | Ref/Alt |  |
| 11 | 17531103 | T | G | *USH1C* | NM_153676:exon18:c.A1813C:p.I605L | Ref/Alt |  |
| 16 | 49672108 | C | T | *ZNF423* | NM_015069:exon4:c.G955A:p.A319T | Ref/Alt | rs199919703 |
|  |  |  |  |  |  |  |  |
| RP#005 |  |  |  |  |  |  |  |
| Chrom | Position | Ref | Alt | Gene Name | Gene Bank ID:Exon:Nucleotide change:Amino Acid change | State | SNP ID |
| 1 | 185962434 | T | G | *HMCN1* | NM_031935:exon23:c.T3498G:p.N1166K | Ref/Alt |  |
| 6 | 80223311 | T | C | *LCA5* | NM_181714:exon4:c.A338G:p.N113S | Ref/Alt | rs181890907 |
| 10 | 55943311 | C | T | *PCDH15* | NM_033056:exon13:c.G1483A:p.V495I | Ref/Alt | rs187727835 |
| 10 | 73550107 | G | A | *CDH23* | NM_022124:exon44:c.G5986A:p.A1996T | Ref/Alt |  |
| 11 | 68207377 | A | C | *LRP5* | NM_002335:exon21:c.A4481C:p.Y1494S | Ref/Alt |  |
| 13 | 114325896 | G | A | *GRK1* | NM_002929:exon3:c.G910A:p.G304S | Ref/Alt |  |
| 14 | 21785998 | C | T | *RPGRIP1* | NM_020366:exon10:c.C1295T:p.S432F | Ref/Alt | rs190985984 |
|  |  |  |  |  |  |  |  |
| RP#006 |  |  |  |  |  |  |  |
| Chrom | Position | Ref | Alt | Gene Name | Gene Bank ID:Exon:Nucleotide change:Amino Acid change | State | SNP ID |
| 1 | 19557394 | A | G | *EMC1* | NM_015047:exon17:c.T2008C:p.F670L | Ref/Alt | rs2275402 |
| 4 | 6302756 | G | C | *WFS1* | NM_006005:exon8:c.G1234C:p.V412L | Ref/Alt | rs149865710 |
| 10 | 73405729 | G | A | *CDH23* | NM_022124:exon12:c.G1282A:p.D428N | Ref/Alt | rs188376296 |
| 12 | 1967743 | C | T | *CACNA2D4* | NM_172364:exon20:c.G2008A:p.G670S | Ref/Alt |  |
| 16 | 28497950 | G | A | *CLN3* | NM_001042432:exon8:c.C482T:p.S161L | Alt/Alt |  |
| 16 | 49672108 | C | T | *ZNF423* | NM_015069:exon4:c.G955A:p.A319T | Ref/Alt | rs199919703 |
|  |  |  |  |  |  |  |  |
| RP#007 |  |  |  |  |  |  |  |
| Chrom | Position | Ref | Alt | Gene Name | Gene Bank ID:Exon:Nucleotide change:Amino Acid change | State | SNP ID |
| 6 | 10784663 | G | C | *MAK* | NM_001242957:exon11:c.C1459G:p.L487V | Ref/Alt |  |
| 8 | 55538842 | A | T | *RP1* | NM_006269:exon4:c.A2400T:p.K800N | Ref/Alt |  |
| 17 | 6406882 | G | A | *PITPNM3* | NM_031220:exon4:c.C239T:p.A80V | Ref/Alt | rs144918108 |
| 17 | 26875038 | C | T | *UNC119* | NM_005148:exon3:c.G416A:p.R139H | Ref/Alt | rs201337554 |
|  |  |  |  |  |  |  |  |
| RP#008 |  |  |  |  |  |  |  |
| Chrom | Position | Ref | Alt | Gene Name | Gene Bank ID:Exon:Nucleotide change:Amino Acid change | State | SNP ID |
| 1 | 19567538 | T | C | *EMC1* | NM_015047:exon6:c.A608G:p.N203S | Ref/Alt |  |
| 1 | 94476477 | G | A | *ABCA4* | NM_000350:exon40:c.C5593T:p.H1865Y | Ref/Alt | rs201707267 |
| 1 | 216348605 | G | A | *USH2A* | NM_206933:exon21:c.C4616T:p.T1539I | Ref/Alt |  |
| 6 | 64791757 | A | G | *EYS* | NM_001142800:exon32:c.T6563C:p.I2188T | Ref/Alt |  |
| 10 | 102770422 | C | T | *PDZD7* | NM_001195263:exon15:c.G2224A:p.A742T | Ref/Alt |  |
| 11 | 17531103 | T | G | *USH1C* | NM_153676:exon18:c.A1813C:p.I605L | Ref/Alt |  |
| 20 | 62626326 | G | T | *PRPF6* | NM_012469:exon5:c.G496T:p.A166S | Ref/Alt |  |
|  |  |  |  |  |  |  |  |
| RP#009 |  |  |  |  |  |  |  |
| Chrom | Position | Ref | Alt | Gene Name | Gene Bank ID:Exon:Nucleotide change:Amino Acid change | State | SNP ID |
| 1 | 103345394 | G | A | *COL11A1* | NM_001854:exon66:c.C5119T:p.R1707W | Ref/Alt |  |
| 4 | 187117196 | A | G | *CYP4V2* | NM_207352:exon3:c.A367G:p.M123V | Ref/Alt | rs149684063 |
| 5 | 89986793 | C | T | *GPR98* | NM_032119:exon31:c.C6886T:p.P2296S | Ref/Alt |  |
| 8 | 68071254 | G | A | *CSPP1* | NM_024790:exon19:c.G2405A:p.R802Q | Ref/Alt |  |
| X | 38158384 | C | T | *RPGR* | NM_000328:exon10:c.G1070A:p.G357D | Alt/Alt |  |
|  |  |  |  |  |  |  |  |
| RP#010 |  |  |  |  |  |  |  |
| Chrom | Position | Ref | Alt | Gene Name | Gene Bank ID:Exon:Nucleotide change:Amino Acid change | State | SNP ID |
| 2 | 110881541 | C | G | *NPHP1* | NM_000272:exon20:c.G2029C:p.E677Q | Ref/Alt |  |
| 4 | 36292017 | G | A | *DTHD1* | NM_001170700:exon2:c.G535A:p.V179I | Ref/Alt | rs200576831 |
| 8 | 38947643 | A | G | *ADAM9* | NM_003816:exon19:c.A2146G:p.I716V | Ref/Alt |  |
| 8 | 68030542 | C | T | *CSPP1* | NM_024790:exon12:c.C1541T:p.P514L | Ref/Alt | rs200535417 |
| 10 | 73545397 | G | A | *CDH23* | NM_022124:exon42:c.G5722A:p.V1908I | Ref/Alt |  |
| 11 | 86662209 | C | T | *FZD4* | NM_012193:exon2:c.G1589A:p.G530E | Ref/Alt | rs201256460 |
| 17 | 79503904 | G | A | *FSCN2* | NM_001077182:exon5:c.G1349A:p.R450H | Ref/Alt |  |
|  |  |  |  |  |  |  |  |
| RP#011 |  |  |  |  |  |  |  |
| Chrom | Position | Ref | Alt | Gene Name | Gene Bank ID:Exon:Nucleotide change:Amino Acid change | State | SNP ID |
| 1 | 215956133 | T | C | *USH2A* | NM_206933:exon53:c.A10532G:p.D3511G | Ref/Alt | rs201997293 |
| 1 | 216496987 | C | T | *USH2A* | NM_206933:exon8:c.G1379A:p.G460E | Ref/Alt |  |
| 2 | 29297043 | G | A | *C2orf71* | NM_001029883:exon1:c.C85T:p.R29W | Ref/Alt | rs201706430 |
| 3 | 63973880 | C | T | *ATXN7* | ATXN7:NM_NM_000333:exon9:c.C1241T:p.P414L | Ref/Alt |  |
| 4 | 39229952 | T | G | *WDR19* | NM_025132:exon16:c.T1752G:p.Y584X | Ref/Alt |  |
| 4 | 39259118 | T | G | *WDR19* | NM_025132:exon28:c.T3127G:p.F1043V | Ref/Alt |  |
| 8 | 10465544 | CG | C | *RP1L1* | NM_178857:exon4:c.6063delC:p.D2021fs | Ref/Alt |  |
| 10 | 73498261 | A | G | *CDH23* | NM_022124:exon33:c.A4216G:p.I1406V | Ref/Alt | rs192459984 |
| 11 | 119215444 | G | A | *MFRP* | NM_031433:exon7:c.C796T:p.R266C | Ref/Alt | rs138295825 |
| 14 | 88897520 | A | G | *SPATA7* | NM_018418:exon9:c.A1033G:p.M345V | Ref/Alt | rs375371982 |
| 16 | 57965705 | T | C | *CNGB1* | NM_001297:exon17:c.A1450G:p.N484D | Ref/Alt |  |
| X | 43817834 | C | T | *NDP* | NM_000266:exon2:c.G58A:p.G20R | Ref/Alt | rs200594881 |
|  |  |  |  |  |  |  |  |
| RP#012 |  |  |  |  |  |  |  |
| Chrom | Position | Ref | Alt | Gene Name | Gene Bank ID:Exon:Nucleotide change:Amino Acid change | State | SNP ID |
| 2 | 29294264 | C | T | *C2orf71* | NM_001029883:exon1:c.G2864A:p.R955Q | Ref/Alt | rs184249075 |
| 4 | 6290790 | T | G | *WFS1* | NM_006005:exon4:c.T392G:p.V131G | Ref/Alt |  |
| 6 | 73043358 | A | G | *RIMS1* | NM_014989:exon29:c.A4186G:p.M1396V | Ref/Alt | rs200994612 |
| 8 | 10465022 | T | C | *RP1L1* | NM_178857:exon4:c.A6586G:p.I2196V | Ref/Alt | rs200407750 |
| 10 | 48389785 | C | T | *RBP3* | NM_002900:exon1:c.G1093A:p.E365K | Ref/Alt |  |
| 15 | 72104355 | C | T | *NR2E3* | NM_014249:exon4:c.C410T:p.S137F | Ref/Alt |  |
|  |  |  |  |  |  |  |  |
| RP#013 |  |  |  |  |  |  |  |
| Chrom | Position | Ref | Alt | Gene Name | Gene Bank ID:Exon:Nucleotide change:Amino Acid change | State | SNP ID |
| 1 | 156145400 | G | A | *SEMA4A* | NM_022367:exon14:c.G1646A:p.S549N | Ref/Alt | rs199696322 |
| 1 | 197396917 | C | T | *CRB1* | NM_201253:exon7:c.C2462T:p.T821M | Ref/Alt | rs142857810 |
| 1 | 216062152 | C | A | *USH2A* | NM_206933:exon41:c.G7839T:p.E2613D | Ref/Alt |  |
| 2 | 73679482 | G | A | *ALMS1* | NM_015120:exon8:c.G5825A:p.R1942H | Ref/Alt | rs146669152 |
| 16 | 1575310 | G | A | *IFT140* | NM_014714:exon22:c.C2786T:p.T929M | Ref/Alt |  |
| X | 49075353 | C | A | *CACNA1F* | NM_005183:exon22:c.G2754T:p.E918D | Alt/Alt |  |
|  |  |  |  |  |  |  |  |
| RP#014 |  |  |  |  |  |  |  |
| Chrom | Position | Ref | Alt | Gene Name | Gene Bank ID:Exon:Nucleotide change:Amino Acid change | State | SNP ID |
| 6 | 65300802 | C | CT | *EYS* | NM_001142800:exon26:c.4957_4958insA:p.S1653fs | Ref/Alt |  |
| 6 | 66205163 | T | A | *EYS* | NM_001142800:exon4:c.A141T:p.E47D | Ref/Alt |  |
| 10 | 126097420 | A | G | *OAT* | NM_000274:exon3:c.T314C:p.V105A | Ref/Alt | rs199957428 |
| 11 | 17531103 | T | G | *USH1C* | NM_153676:exon18:c.A1813C:p.I605L | Ref/Alt |  |
| 11 | 66282046 | C | A | *BBS1* | NM_024649:exon4:c.C329A:p.P110H | Ref/Alt |  |
| 16 | 57921800 | C | T | *CNGB1* | NM_001297:exon32:c.G3421A:p.A1141T | Ref/Alt | rs201449358 |
|  |  |  |  |  |  |  |  |
| RP#015 |  |  |  |  |  |  |  |
| Chrom | Position | Ref | Alt | Gene Name | Gene Bank ID:Exon:Nucleotide change:Amino Acid change | State | SNP ID |
| 6 | 72892840 | G | T | *RIMS1* | NM_014989:exon6:c.G1666T:p.V556F | Ref/Alt |  |
| 8 | 10464767 | G | T | *RP1L1* | NM_178857:exon4:c.C6841A:p.P2281T | Ref/Alt |  |
| 8 | 55538461 | G | GA | *RP1* | NM_006269:exon4:c.2020dupA:p.K673fs | Ref/Alt |  |
| 9 | 2718241 | G | A | *KCNV2* | NM_133497:exon1:c.G502A:p.G168R | Ref/Alt |  |
| 10 | 73569606 | G | A | *CDH23* | NM_022124:exon59:c.G8752A:p.D2918N | Ref/Alt |  |
| 10 | 102770422 | C | T | *PDZD7* | NM_001195263:exon15:c.G2224A:p.A742T | Ref/Alt |  |
| 20 | 10393406 | A | G | *MKKS* | NM_018848:exon3:c.T757C:p.S253P | Ref/Alt | rs201785599 |
|  |  |  |  |  |  |  |  |
| RP#016 |  |  |  |  |  |  |  |
| Chrom | Position | Ref | Alt | Gene Name | Gene Bank ID:Exon:Nucleotide change:Amino Acid change | State | SNP ID |
| 6 | 35471413 | G | A | *TULP1* | NM_003322:exon13:c.C1246T:p.R416C | Ref/Alt | rs200769197 |
| 6 | 35480633 | C | T | *TULP1* | NM_003322:exon1:c.G3A:p.M1I | Ref/Alt |  |
| 6 | 76660583 | C | T | *IMPG1* | NM_001563:exon13:c.G1520A:p.R507Q | Ref/Alt |  |
| 10 | 73545397 | G | A | *CDH23* | NM_022124:exon42:c.G5722A:p.V1908I | Ref/Alt |  |
| 20 | 10621771 | T | A | *JAG1* | NM_000214:exon24:c.A3038T:p.H1013L | Ref/Alt |  |
|  |  |  |  |  |  |  |  |
| RP#017 |  |  |  |  |  |  |  |
| Chrom | Position | Ref | Alt | Gene Name | Gene Bank ID:Exon:Nucleotide change:Amino Acid change | State | SNP ID |
| 2 | 63631776 | C | T | *WDPCP* | NM_015910:exon10:c.G842A:p.R281H | Ref/Alt | rs3738877 |
| 2 | 99012501 | C | T | *CNGA3* | NM_001298:exon8:c.C868T:p.R290C | Ref/Alt | rs367597798 |
| 5 | 82835958 | A | T | *VCAN* | NM_004385:exon8:c.A7136T:p.E2379V | Ref/Alt | rs201642515 |
| 6 | 65300802 | C | CT | *EYS* | NM_001142800:exon26:c.4957_4958insA:p.S1653fs | Ref/Alt |  |
| 6 | 65301737 | AG | A | *EYS* | NM_001142800:exon26:c.4022delC:p.S1341fs | Ref/Alt |  |
| 8 | 87679304 | C | A | *CNGB3* | NM_019098:exon6:c.G701T:p.C234F | Ref/Alt |  |
|  |  |  |  |  |  |  |  |
| RP#018 |  |  |  |  |  |  |  |
| Chrom | Position | Ref | Alt | Gene Name | Gene Bank ID:Exon:Nucleotide change:Amino Acid change | State | SNP ID |
| 3 | 150659395 | C | T | *CLRN1* | NM_174878:exon2:c.G407A:p.G136E | Ref/Alt |  |
| 6 | 42666119 | A | G | *PRPH2* | NM_000322:exon3:c.T955C:p.F319L | Ref/Alt | rs139329966 |
| 11 | 61727452 | C | A | *BEST1* | NM_004183:exon9:c.C1037A:p.P346H | Ref/Alt |  |
| 14 | 89338734 | A | C | *TTC8* | NM_198309:exon12:c.A1255C:p.N419H | Ref/Alt |  |
|  |  |  |  |  |  |  |  |
| RP#019 |  |  |  |  |  |  |  |
| Chrom | Position | Ref | Alt | Gene Name | Gene Bank ID:Exon:Nucleotide change:Amino Acid change | State | SNP ID |
| 1 | 196695677 | C | T | *CFH* | NM_000186:exon13:c.C1951T:p.H651Y | Ref/Alt |  |
| 1 | 216373302 | T | C | *USH2A* | NM_206933:exon17:c.A3478G:p.I1160V | Ref/Alt |  |
| 2 | 73679482 | G | A | *ALMS1* | NM_015120:exon8:c.G5825A:p.R1942H | Ref/Alt | rs146669152 |
| 4 | 47939081 | AC | A | *CNGA1* | NM_000087:exon11:c.1429delG:p.V477fs | Ref/Alt |  |
| 4 | 47951883 | AG | A | *CNGA1* | NM_000087:exon6:c.265delC:p.L89fs | Ref/Alt |  |
| 5 | 82836105 | C | T | *VCAN* | NM_004385:exon8:c.C7283T:p.P2428L | Ref/Alt |  |
| 16 | 53692703 | C | T | *RPGRIP1L* | NM_015272:exon11:c.G1331A:p.R444H | Ref/Alt | rs76600508 |
| 20 | 3891375 | A | G | *PANK2* | NM_153638:exon3:c.A1133G:p.D378G | Ref/Alt |  |
|  |  |  |  |  |  |  |  |
| RP#020 |  |  |  |  |  |  |  |
| Chrom | Position | Ref | Alt | Gene Name | Gene Bank ID:Exon:Nucleotide change:Amino Acid change | State | SNP ID |
| 1 | 5927122 | C | T | *NPHP4* | NM_015102:exon25:c.G3526A:p.D1176N | Ref/Alt |  |
| 4 | 16010674 | A | G | *PROM1* | NM_006017:exon11:c.T1199C:p.L400P | Ref/Alt | rs140682455 |
| 4 | 22390013 | G | A | *GPR125* | NM_145290:exon19:c.C3281T:p.A1094V | Ref/Alt | rs141619991 |
| 6 | 64431122 | G | T | *EYS* | NM_001142800:exon43:c.C8805A:p.Y2935X | Ref/Alt |  |
| 7 | 92147204 | T | G | *PEX1* | NM_000466:exon5:c.A625C:p.M209L | Ref/Alt |  |
| 11 | 68205950 | A | C | *LRP5* | NM_002335:exon20:c.A4148C:p.H1383P | Ref/Alt |  |
| 15 | 31362068 | C | A | *TRPM1* | NM_001252030:exon3:c.G379T:p.A127S | Ref/Alt | rs140493891 |
| 16 | 16248624 | G | A | *ABCC6* | NM_001171:exon29:c.C4069T:p.R1357W | Ref/Alt | rs63750428 |
|  |  |  |  |  |  |  |  |
| RP#021 |  |  |  |  |  |  |  |
| Chrom | Position | Ref | Alt | Gene Name | Gene Bank ID:Exon:Nucleotide change:Amino Acid change | State | SNP ID |
| 2 | 110886786 | G | T | *NPHP1* | NM_000272:exon18:c.C1861A:p.P621T | Ref/Alt | rs147090619 |
| 4 | 47953414 | AC | A | *CNGA1* | NM_000087:exon5:c.191delG:p.G64fs | Alt/Alt |  |
| 5 | 82835958 | A | T | *VCAN* | NM_004385:exon8:c.A7136T:p.E2379V | Ref/Alt | rs201642515 |
| 5 | 90124929 | G | A | *GPR98* | NM_032119:exon77:c.G16537A:p.A5513T | Ref/Alt |  |
| 6 | 76657075 | T | C | *IMPG1* | NM_001563:exon14:c.A2000G:p.Q667R | Alt/Alt |  |
| 7 | 120478842 | G | A | *TSPAN12* | NM_012338:exon4:c.C274T:p.L92F | Ref/Alt |  |
| 17 | 26875038 | C | T | *UNC119* | NM_005148:exon3:c.G416A:p.R139H | Ref/Alt | rs201337554 |
| 17 | 72916678 | C | T | *USH1G* | NM_173477:exon2:c.G253A:p.V85M | Ref/Alt |  |
| 20 | 3870130 | G | A | *PANK2* | NM_153638:exon1:c.G383A:p.R128Q | Ref/Alt |  |
|  |  |  |  |  |  |  |  |
| RP#022 |  |  |  |  |  |  |  |
| Chrom | Position | Ref | Alt | Gene Name | Gene Bank ID:Exon:Nucleotide change:Amino Acid change | State | SNP ID |
| 1 | 94471025 | C | T | *ABCA4* | NM_000350:exon44:c.G6119A:p.R2040Q | Ref/Alt | rs148460146 |
| 1 | 216062152 | C | A | *USH2A* | NM_206933:exon41:c.G7839T:p.E2613D | Ref/Alt |  |
| 6 | 65300802 | C | CT | *EYS* | NM_001142800:exon26:c.4957_4958insA:p.S1653fs | Ref/Alt |  |
| 10 | 55582297 | A | T | *PCDH15* | NM_033056:exon33:c.T5189A:p.I1730N | Ref/Alt |  |
| 11 | 8115692 | G | A | *TUB* | NM_003320:exon5:c.G518A:p.G173D | Ref/Alt | rs139734163 |
| 14 | 21813310 | C | T | *RPGRIP1* | NM_020366:exon22:c.C3571T:p.R1191W | Ref/Alt | rs188660364 |
| 15 | 31327796 | T | C | *TRPM1* | NM_002420:exon20:c.A2587G:p.I863V | Ref/Alt | rs201650867 |
| 17 | 6374504 | A | T | *PITPNM3* | NM_031220:exon12:c.T1601A:p.M534K | Ref/Alt |  |
|  |  |  |  |  |  |  |  |
| RP#023 |  |  |  |  |  |  |  |
| Chrom | Position | Ref | Alt | Gene Name | Gene Bank ID:Exon:Nucleotide change:Amino Acid change | State | SNP ID |
| 1 | 215953265 | A | G | *USH2A* | NM_206933:exon55:c.T10859C:p.I3620T | Ref/Alt |  |
| 1 | 215987141 | G | A | *USH2A* | NM_206933:exon49:c.C9676T:p.R3226X | Ref/Alt |  |
| 5 | 90445941 | G | A | *GPR98* | NM_032119:exon88:c.G18527A:p.G6176E | Ref/Alt |  |
| 5 | 149264115 | G | A | *PDE6A* | NM_000440:exon16:c.C1954T:p.R652C | Ref/Alt | rs199748187 |
| 6 | 65336128 | C | T | *EYS* | NM_001142800:exon23:c.G3454A:p.G1152R | Ref/Alt | rs371491059 |
| 6 | 76728469 | T | C | *IMPG1* | NM_001563:exon7:c.A773G:p.Y258C | Ref/Alt |  |
| 8 | 10465544 | CG | C | *RP1L1* | NM_178857:exon4:c.6063delC:p.D2021fs | Ref/Alt |  |
| 8 | 68071254 | G | A | *CSPP1* | NM_024790:exon19:c.G2405A:p.R802Q | Ref/Alt |  |
| 10 | 27493409 | C | A | *ACBD5* | NM_145698:exon12:c.G1525T:p.A509S | Ref/Alt |  |
| 11 | 17531103 | T | G | *USH1C* | NM_153676:exon18:c.A1813C:p.I605L | Ref/Alt |  |
|  |  |  |  |  |  |  |  |
| RP#024 |  |  |  |  |  |  |  |
| Chrom | Position | Ref | Alt | Gene Name | Gene Bank ID:Exon:Nucleotide change:Amino Acid change | State | SNP ID |
| 1 | 19571465 | T | C | *EMC1* | NM_015047:exon2:c.A155G:p.K52R | Ref/Alt | rs201537299 |
| 1 | 94544208 | C | T | *ABCA4* | NM_000350:exon10:c.G1294A:p.E432K | Ref/Alt | rs201117452 |
| 4 | 13371588 | C | T | *RAB28* | NM_004249:exon7:c.G575A:p.R192H | Ref/Alt |  |
| 4 | 39188196 | CA | C | *WDR19* | NM_025132:exon3:c.137delA:p.Q46fs | Ref/Alt |  |
| 5 | 89925321 | G | A | *GPR98* | NM_032119:exon9:c.G1804A:p.A602T | Ref/Alt | rs201015784 |
| 6 | 65300335 | A | G | *EYS* | NM_001142800:exon26:c.T5425C:p.S1809P | Ref/Alt |  |
| 12 | 88523587 | A | G | *CEP290* | NM_025114:exon10:c.T736C:p.S246P | Ref/Alt |  |
| 15 | 89755011 | C | T | *RLBP1* | NM_000326:exon7:c.G647A:p.R216Q | Ref/Alt | rs200488706 |
| X | 49084807 | T | C | *CACNA1F* | NM_005183:exon7:c.A920G:p.N307S | Alt/Alt |  |
|  |  |  |  |  |  |  |  |
| RP#025 |  |  |  |  |  |  |  |
| Chrom | Position | Ref | Alt | Gene Name | Gene Bank ID:Exon:Nucleotide change:Amino Acid change | State | SNP ID |
| 2 | 96949315 | A | G | *SNRNP200* | NM_014014:exon33:c.T4721C:p.I1574T | Ref/Alt | rs199736893 |
| 4 | 6290790 | T | G | *WFS1* | NM_006005:exon4:c.T392G:p.V131G | Ref/Alt |  |
| 6 | 73001695 | C | T | *RIMS1* | NM_014989:exon26:c.C3796T:p.R1266C | Ref/Alt |  |
| 8 | 96272735 | T | C | *C8orf37* | NM_177965:exon3:c.A269G:p.N90S | Ref/Alt | rs199731969 |
|  |  |  |  |  |  |  |  |
| RP#026 |  |  |  |  |  |  |  |
| Chrom | Position | Ref | Alt | Gene Name | Gene Bank ID:Exon:Nucleotide change:Amino Acid change | State | SNP ID |
| 1 | 19567602 | CG | C | *EMC1* | NM_015047:exon6:c.543delC:p.Y181X | Ref/Alt |  |
| 1 | 68910495 | C | T | *RPE65* | NM_000329:exon4:c.G317A:p.C106Y | Ref/Alt | rs142052358 |
| 1 | 185946967 | G | A | *HMCN1* | NM_031935:exon16:c.G2420A:p.G807D | Ref/Alt |  |
| 1 | 215933099 | C | T | *USH2A* | NM_206933:exon57:c.G11134A:p.V3712I | Ref/Alt | rs201951918 |
| 1 | 216465520 | T | C | *USH2A* | NM_206933:exon10:c.A1837G:p.T613A | Ref/Alt |  |
| 5 | 89925321 | G | A | *GPR98* | NM_032119:exon9:c.G1804A:p.A602T | Ref/Alt | rs201015784 |
| 6 | 65300802 | C | CT | *EYS* | NM_001142800:exon26:c.4957_4958insA:p.S1653fs | Alt/Alt |  |
| 9 | 2718448 | C | T | *KCNV2* | NM_133497:exon1:c.C709T:p.R237C | Ref/Alt |  |
| 15 | 31294573 | G | C | *TRPM1* | NM_002420:exon27:c.C4264G:p.R1422G | Ref/Alt | rs3784587 |
|  |  |  |  |  |  |  |  |
| RP#027 |  |  |  |  |  |  |  |
| Chrom | Position | Ref | Alt | Gene Name | Gene Bank ID:Exon:Nucleotide change:Amino Acid change | State | SNP ID |
| 1 | 94544208 | C | T | *ABCA4* | NM_000350:exon10:c.G1294A:p.E432K | Ref/Alt | rs201117452 |
| 1 | 216420322 | C | G | *USH2A* | NM_206933:exon13:c.G2414C:p.G805A | Ref/Alt |  |
| 2 | 29294633 | G | A | *C2orf71* | NM_001029883:exon1:c.C2495T:p.P832L | Ref/Alt |  |
| 2 | 234243724 | CA | C | *SAG* | NM_000541:exon11:c.924delA:p.T308fs | Alt/Alt |  |
| 5 | 82833933 | A | G | *VCAN* | NM_004385:exon8:c.A5111G:p.K1704R | Ref/Alt |  |
| 8 | 96272735 | T | C | *C8orf37* | NM_177965:exon3:c.A269G:p.N90S | Ref/Alt | rs199731969 |
| 10 | 73572631 | G | T | *CDH23* | NM_022124:exon66:c.G9617T:p.R3206L | Ref/Alt |  |
| 17 | 36485943 | C | T | *GPR179* | NM_001004334:exon11:c.G3509A:p.R1170Q | Ref/Alt |  |
|  |  |  |  |  |  |  |  |
| RP#028 |  |  |  |  |  |  |  |
| Chrom | Position | Ref | Alt | *Gene Name* | Gene Bank ID:Exon:Nucleotide change:Amino Acid change | State | SNP ID |
| 1 | 215807920 | A | G | *USH2A* | NM_206933:exon70:c.T15178C:p.S5060P | Ref/Alt |  |
| 1 | 215953193 | G | A | *USH2A* | NM_206933:exon55:c.C10931T:p.T3644M | Alt/Alt | rs185823130 |
| 1 | 216062111 | A | G | *USH2A* | NM_206933:exon41:c.T7880C:p.I2627T | Ref/Alt |  |
| 2 | 112786150 | TGCCTCC | T | *MERTK* | NM_006343:exon19:c.2710_2715del:p.904_905del | Ref/Alt |  |
| 6 | 42666119 | A | G | *PRPH2* | NM_000322:exon3:c.T955C:p.F319L | Ref/Alt | rs139329966 |
| 7 | 138602379 | G | A | *KIAA1549* | NM_001164665:exon2:c.C1993T:p.P665S | Ref/Alt |  |
| 10 | 73498373 | C | G | *CDH23* | NM_022124:exon33:c.C4328G:p.A1443G | Ref/Alt |  |
| 11 | 76873191 | G | A | *MYO7A* | NM_000260:exon13:c.G1369A:p.A457T | Ref/Alt |  |
| 16 | 16291933 | T | C | *ABCC6* | NM_001171:exon10:c.A1283G:p.N428S | Ref/Alt | rs201880691 |
| 17 | 79495808 | C | T | *FSCN2* | NM_001077182:exon1:c.C251T:p.P84L | Ref/Alt | rs373682711 |
|  |  |  |  |  |  |  |  |
| RP#029 |  |  |  |  |  |  |  |
| Chrom | Position | Ref | Alt | Gene Name | Gene Bank ID:Exon:Nucleotide change:Amino Acid change | State | SNP ID |
| 1 | 186043883 | C | T | *HMCN1* | NM_031935:exon53:c.C8150T:p.S2717F | Ref/Alt | rs201586866 |
| 1 | 186151336 | A | G | *HMCN1* | NM_031935:exon105:c.A16331G:p.H5444R | Ref/Alt |  |
| 4 | 47951883 | AG | A | *CNGA1* | NM_000087:exon6:c.265delC:p.L89fs | Alt/Alt |  |
| 6 | 76751727 | C | T | *IMPG1* | NM_001563:exon2:c.G184A:p.D62N | Ref/Alt | rs137915302 |
| 9 | 2718871 | G | T | *KCNV2* | NM_133497:exon1:c.G1132T:p.V378F | Ref/Alt |  |
| 17 | 56289785 | C | T | *MKS1* | NM_017777:exon9:c.G869A:p.R290Q | Ref/Alt |  |
|  |  |  |  |  |  |  |  |
| RP#030 |  |  |  |  |  |  |  |
| Chrom | Position | Ref | Alt | Gene Name | Gene Bank ID:Exon:Nucleotide change:Amino Acid change | State | SNP ID |
| 2 | 29287854 | G | A | *C2orf71* | NM_001029883:exon2:c.C3748T:p.R1250C | Ref/Alt |  |
| 2 | 29297043 | G | A | *C2orf71* | NM_001029883:exon1:c.C85T:p.R29W | Ref/Alt | rs201706430 |
| 2 | 73680229 | G | A | *ALMS1* | NM_015120:exon8:c.G6572A:p.G2191D | Ref/Alt | rs193286818 |
| 4 | 22390493 | T | C | *GPR125* | NM_145290:exon19:c.A2801G:p.Y934C | Ref/Alt | rs137992274 |
| 4 | 100503124 | G | A | *MTTP* | NM_000253:exon3:c.G124A:p.V42I | Ref/Alt |  |
| 4 | 187126358 | A | C | *CYP4V2* | NM_207352:exon8:c.A992C:p.H331P | Ref/Alt | rs199476197 |
| 6 | 64430739 | CTG | C | *EYS* | NM_001142800:exon43:c.9186_9187del:p.3062_3063del | Ref/Alt |  |
| 8 | 55542239 | C | T | *RP1* | NM_006269:exon4:c.C5797T:p.R1933X | Ref/Alt | rs118031911 |
| 10 | 85955300 | A | G | *CDHR1* | NM_033100:exon2:c.A106G:p.N36D | Ref/Alt | rs200661366 |
| 10 | 86008715 | G | A | *RGR* | NM_001012720:exon3:c.G274A:p.G92S | Ref/Alt |  |
| 16 | 57918336 | C | T | *CNGB1* | NM_001297:exon33:c.G3488A:p.G1163E | Ref/Alt | rs186853874 |
| 17 | 6387591 | A | G | *PITPNM3* | NM_031220:exon5:c.T296C:p.L99S | Ref/Alt |  |
| 17 | 63193275 | C | T | *RGS9* | NM_003835:exon13:c.C892T:p.R298X | Ref/Alt |  |

Chrom = chromosome, Ref = reference allele, Alt = alternative allele

**Supplemental Table S2.**

***CNGA1* primers and PCR conditions**

*When Sanger sequencing reaction is performed for Exon 11d, the primer CNGA1-11dR (TTTGTGGTATTGAGCGATGG) is used rather than the primer CNGA1-11cR.

| Region to amplify | Primer name | Sequence (5´ to 3´) | Annealing temperature |
| --- | --- | --- | --- |
| Exon 4 | CNGA1-4F | GACAAGTTATGCAGTTCCAAAG | 56ºC |
|  | CNGA1-4R | TTCATCTTAAAGTGCGGCTC |  |
| Exon 5 | CNGA1-5F | AATTCCTCCTGAGATCCCAC | 56ºC |
|  | CNGA1-5R | CTCTCTCTGCCTCCTATTGG |  |
| Exon 6 | CNGA1-6F | TCACAGAGAAAGTCAACTGC | 56ºC |
|  | CNGA1-6R | CAGACCATTTGCCTACCATG |  |
| Exon 7-8 | CNGA1-7-8F | ATCTTCACCAATGCTTAGGG | 56ºC |
|  | CNGA1-7-8R | CTGGAGCAGGTCTTTGAATG |  |
| Exon 9 | CNGA1-9F | ACCTGCAGTAGAGAAAGGAG | 62ºC |
|  | CNGA1-9R | TTGTGGTTGATGGGCATAAC |  |
| Exon 10 | CNGA1-10F | CACAAAGCTGAGTTATGTGC | 58ºC |
|  | CNGA1-10R | AAGGACTCTAAGGACCTCTC |  |
| Exon 11a | CNGA1-11aF | AAACGGCCAAATTCAGGATC | 56ºC |
|  | CNGA1-11aR | TGATATTGGGTCTCAGCCTC |  |
| Exon 11b | CNGA1-11bF | TCGCTCAATACCACAAACTG | 56ºC |
|  | CNGA1-11bR | ACTGGAATGCATGTGTGTTC |  |
| Exon 11c | CNGA1-11cF | CATACTCAGCCAAGATTCGG | 62ºC |
|  | CNGA1-11cR | TTGACTACCTGTGGACCAAC |  |
| Exon 11d | CNGA1-11dF | CCTTGTACCTTGCACCAAAG | 60ºC |
|  | CNGA1-11cR* | TTGACTACCTGTGGACCAAC |  |

**Supplemental Table S3.**

**Haplotype analysis of four patients with retinitis pigmentosa with *CNGA1* mutations**

| Chrom | Position | SNP ID | Ref | Alt | RP#002 | RP#019 | RP#021 | RP#029 | Frequency* |
| --- | --- | --- | --- | --- | --- | --- | --- | --- | --- |
| 4 | 47,839,929 | rs2289433 | C | T | Alt/Alt | Ref/Alt | Alt/Alt | Ref/Ref | 0.3202 |
| 4 | 47,844,064 | rs56001837 | G | T | Alt/Alt | Ref/Alt | Alt/Alt | Ref/Ref | 0.3652 |
| 4 | 47,853,843 | rs6822668 | T | C | Alt/Alt | Ref/Alt | Alt/Alt | Ref/Ref | 0.3202 |
| 4 | 47,880,494 | rs2289435 | G | A | Alt/Alt | Ref/Alt | Alt/Alt | Ref/Ref | 0.3989 |
| 4 | 47,886,319 | rs12510002 | A | C | Alt/Alt | Ref/Alt | Alt/Alt | Ref/Ref | 0.3652 |
| 4 | 47,887,536 | rs2053404 | T | C | Alt/Alt | Ref/Alt | Alt/Alt | Ref/Ref | 0.3202 |
| 4 | 47,887,991 | rs6818556 | G | A | Alt/Alt | Ref/Alt | Alt/Alt | Ref/Ref | 0.3202 |
| 4 | 47,898,491 | rs6447592 | C | T | Alt/Alt | Ref/Alt | Alt/Alt | Ref/Ref | 0.3202 |
| 4 | 47,901,476 | rs12651301 | G | A | Alt/Alt | Ref/Alt | Alt/Alt | Ref/Ref | 0.3652 |
| 4 | 47,945,295 | rs28642966 | C | T | Alt/Alt | Ref/Ref | Alt/Alt | Ref/Ref | 0.3989 |
| 4 | 48,018,529 | rs321633 | T | C | Ref/Ref | Alt/Alt | Ref/Ref | Alt/Alt | 0.4045 |
| 4 | 48,018,614 | rs321634 | C | G | Ref/Ref | Ref/Alt | Ref/Ref | Ref/Ref | 0.264 |
| 4 | 48,018,761 | rs77335151 | A | G | Ref/Ref | Ref/Alt | Ref/Ref | Alt/Alt | 0.0506 |
| 4 | 48,037,926 | rs13116684 | A | G | Ref/Ref | Ref/Alt | Ref/Ref | Ref/Ref | 0.264 |
| 4 | 48,038,379 | rs10805163 | C | T | Ref/Ref | Alt/Alt | Ref/Ref | Alt/Alt | 0.4045 |
| 4 | 48,038,716 | rs11722463 | G | A | Ref/Ref | Alt/Alt | Ref/Ref | Alt/Alt | 0.3146 |
| 4 | 48,039,670 | rs10938520 | A | G | Ref/Ref | Alt/Alt | Ref/Ref | Alt/Alt | 0.3933 |
| 4 | 48,039,902 | rs11736427 | A | T | Ref/Ref | Alt/Alt | Ref/Ref | Alt/Alt | 0.3146 |
| 4 | 48,068,783 | rs1567217 | G | A | Ref/Ref | Ref/Alt | Ref/Ref | Ref/Ref | 0.2528 |
| 4 | 48,069,696 | rs2230594 | G | C | Alt/Alt | Ref/Ref | Alt/Alt | Ref/Ref | 0.4663 |
| 4 | 48,069,725 | rs9996535 | G | A | Alt/Alt | Ref/Ref | Alt/Alt | Ref/Ref | 0.4663 |

Chrom = Chromosome, Ref = reference allele, Alt = Alternative allele, Frequency* indicates the frequency in Japanese individuals and is obtained from the 1000 Genomes project (http://www.1000genomes.org)
